# Removal of Cd2+ metal ions from aqueous solutions by Na-alginate-containing composite biosorbent

**DOI:** 10.55730/1300-0527.3365

**Published:** 2022-01-11

**Authors:** Ramazan DONAT

**Affiliations:** Pamukkale University, Faculty of Sciences Arts, Department of Chemistry, Denizli, Turkey

**Keywords:** Cadmium, Na-alginate, composite biosorbent, sorption isotherms

## Abstract

This research is related to the effect of alginate-containing composite biosorbent on the sorption capacity of heavy metal Cd^2+^ ions. The study aims to determine the character and optimization of the sorption of Na-alginate-containing composite biosorbent on the sorption of heavy metal Cd^2+^ metal ions in an aqueous solution. Optimum conditions of parameters such as biosorbent amount, pH value effect, concentration effect, contact time, and temperature that affect the sorption of Cd^2+^ ions were determined. Langmuir, Freundlich, D-R isotherms were applied to the obtained data, and it was noted that the sorption Cd^2+^ ions are better explained with Freundlich and D-R models. The thermodynamic parameters (Δ*G^o^*, *ΔH*^o^, *ΔS*^o^) for Cd^2+^ sorption onto composite biosorbent were also determined given from the temperature dependence. The results suggested that composite biosorbent is suitable as a sorbent material for recovery and sorption of metal ions from aqueous solutions.

## 1. Introduction

Environmental pollution by cadmium is caused by waste from various industrial activities including the chemical, ceramics, galvanizing, and textile industries, which are potential sources of water pollution by cadmium ions. Cadmium ions from heavy metal ions cause acute toxicity on the human body, as well as diseases such as lung cancer and kidney failure [1].

Contamination of various heavy metals is considered an environmental problem that has a negative impact on human and health ecosystems, one of which is metal cadmium (Cd). One of the wastes that pollute the environment is liquid waste containing heavy metals, which have bad effects on living things and the environment [2].

Heavy metals are the types of pollutants that are very harmful to environmental systems because they are nonbiodegradable, toxic, and capable of bioaccumulation in the food chain [3]. One example of a very dangerous heavy metal is metal cadmium (Cd). Cadmium is one of the metals that are grouped by type of non-essential heavy metals. Pollution by cadmium also has a negative impact on ecosystems and human life. Cadmium when accumulated in the body in the long term can interfere with the reproductive system, kidneys and also damage the nervous system [4].

Therefore, we need a way to reduce the levels of heavy metals, especially in the waters. Some techniques used to reduce cadmium levels in water include precipitation, coagulation, ion exchange, electrochemistry and separation using membranes, but these methods have several drawbacks, including the use of a lot of raw materials and require a lot of energy, less effective, especially in metal treatment with low concentrations [5]. Cadmium in water sources tends to be present in very low levels (trace metals). Therefore, it is necessary to have certain techniques in determining the concentration of these metals, one of which is preconcentration.

Many techniques and methods such as neutralization, biosorption, precipitation, ion exchange, electrolysis, and adsorption have been developed to remove heavy metal ions from water [6]. Among these methods, the adsorption technique is the most effective way to remove heavy metal ions from water due to its low cost and simple application technique [7].

In the adsorption process, adsorbents which are generally porous materials that have certain groups that can bind to metals are used one of the adsorbents is alginate. Alginate is one type of natural polymer obtained from the extraction process of brown seaweed, which contains polysaccharides on the cell wall in the form of alginic acid, which is easily obtained and biocompatible [8]. Alginate has a carboxylic acid functional group which is an active compound capable of bonding with a metal [9]. Carboxyl in alginate with multivalent cation Ca^2+^ allows gel formation to occur. Cations other than Ca^2+^ cannot be used because they are less stable. Calcium is the most widely one generally due to its ability to form a stable gel with alginate, its low cost, its easy availability, and its nontoxic nature. The formation of this gel can also increase the absorption ability because it has a large pore volume compared to the form of flakes, so this alginate is very effective for the metal adsorption process through the presence of coupling with carbon nanotubes, as well as those modified with chelating agents [5,8,9].

In this study, the sorption ability of cadmium metal ions in aqueous solution of composite biosorbent containing alginate was investigated as a function of some experimental parameters. According to the data obtained, the sorption of Cd^2+^ metal ions onto the composite biosorbent was evaluated by calculating the suitability for three different adsorption isotherms and thermodynamic parameters. The sorption processes based on this alginate-containing composite biosorbent represent a possible alternative to the conventional cadmium separation and purification processes.

## 2. Materials and methods

### 2.1. Preparation of composite biosorbent containing Na-alginate

**Figure 1:**
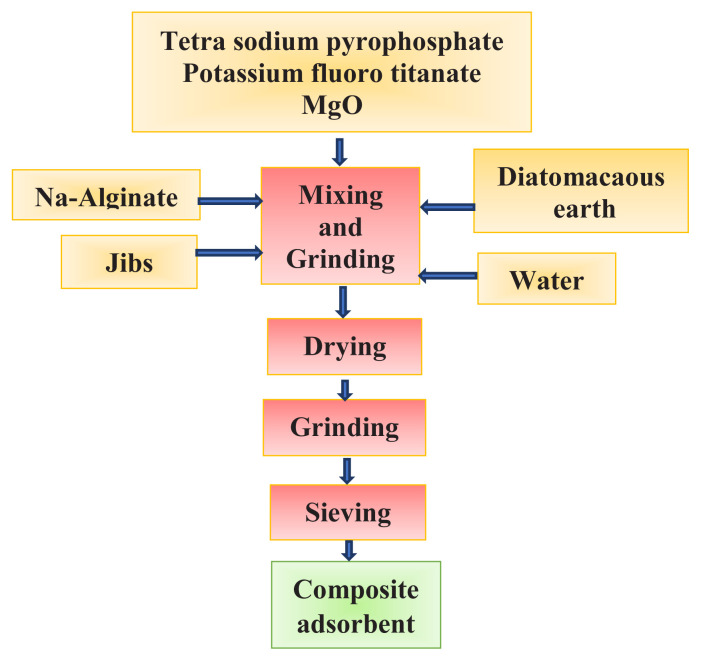
Flow sheet for the preparation of alginate-containing composite biosorbent.

Composite adsorbent of various concentrations was prepared by mixing the necessary amounts of solid materials (Na-alginate, gypsum, diatomite, magnesium oxide, potassium fluoro titanate, tetrasodium pyrophosphate) and distilled water. The percent of mixing ratios of the composite biosorbent composition is given in Table 1. The schematic diagram of the general process steps in the preparation of the composite adsorbent is shown in Figure 1.

The composite biosorbent given in Table 1 is the formulation of the dental impression (alginate impression material) material. Particularly alginate used in formulation is used in almost all branches of dentistry, which functions to create an impression of the relationship between teeth and oral tissues (hard tissue and soft tissue) to obtain negative impressions or models of oral tissue. It is known that the lack of mechanical properties of alginate is improved with the right amount of filler.

As seen in Figure 1, distilled water was used to provide a homogeneous mixture in the process steps to form the composite biosorbent, and then it was dried in an oven at 100±5 ^o^C. The dry composite biosorbent was ground and sieved with a particle size of 125 microns. Then, it was preserved in an air and moisture-proof glass jar.

### 2.2. Determination of Cd^2+^ metal ions in solution

**Figure 2:**
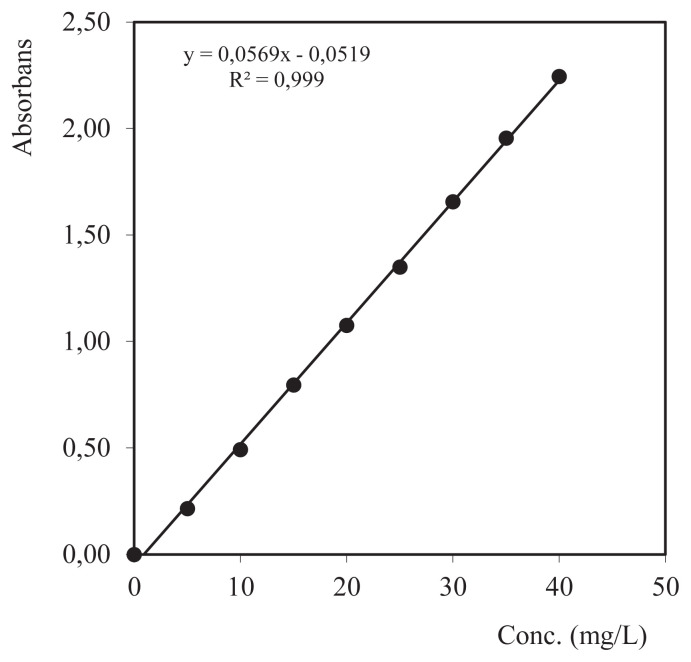
Cd^2+^ metal ions standard calibration curve in the concentration range 0–40 mg/L.

In determining the concentration of Cd^2+^ metal ions that are not absorbed by the composite adsorbent, a UV spectrophotometer (dithizone-λ= 600 nm) is used in the measurement so that a calibration curve is made to find the linearity area of measurement between the analyte concentration in the sample and the given measuring area [10]. The linearity of the graph is obtained by plotting the absorbance values as a function of the analyte concentration, which is generally called the calibration curve. According to the measurement results, it can be seen from Figure 2 that the regression line equation y = 0.0569×–0.0519 (*R*^2^= 0.999).

### 2.3. Data analysis

From each ratio of the analysis data, a graph of the absorption of Cd^2+^ metal ions was prepared. Furthermore, from the results obtained, the % sorption capacity (*Q*_e_) was determined as well as the biosorption isotherm analysis. Cheragi et al. [11] reported that % sorption capacity (*Q*_e_) was calculated using the Eq. (1) [11,12]:


(1) $$Q_{e}=\frac{(C_{0}-C_{e})v}{m}$$

Where *Q*_e_: sorption capacity (mg/g), *m*: mass of biosorbent (grams), *v*: volume of solution (mL), *C*_o_: initial concentration before sorption (mg/L), *C*e: equilibrium concentration of Cd^2+^ ion sorption (mg/L).

The percent sorption (%) and distribution constant (*K*_d_) were calculated using the following Eqs. (2), (3) [13]:


(2) $$\%\;Removal=\frac{\left(C_{o}-C_{e}\right)}{C_{o}}\times 100$$

where *C*_o_ and *C*_e_ are the initial concentration and equilibrium concentration of the Cd^2+^ ion solution (mg/L), respectively.


(3) $$K_{d}=\frac{C_{init.}-C_{eq.}}{C_{eq.}}\frac{V}{m}$$

Where *C*_init._ and *C*_eq_ are the initial and the equilibrium concentrations of Cd^2+^ metal ions in solution, respectively. *V*: the volume of liquid phase (mL), *m*: the weight of the biosorbent (g).

## 3. Results and discussions

### 3.1 Effect of pH value

pH value is a very important parameter, which can affect the functional groups that play an active role in the cell wall of biomass in the absorption of heavy metals. Besides having an effect on functional groups, pH value can also affect the solubility of metal ions in the solution. Determination of pH value variation is done to determine the pH value of the interaction where the adsorbent absorbs heavy metals optimally.

The metal ion Cd^2+^ with a concentration of 100 mg/L as the initial concentration (*C*_o_) was added to a 10 mL plastic tube. The pH value of the solution was adjusted to 3, 4, 5, 6, and 7 respectively, using 0.10 M HNO_3_ and 0.10 M NaOH. Then, 0.10 g of composite biosorbent was added, and the mixture was shaken using a shaker at 60 rpm for 15 min. After contacting, the mixture was filtered, and the filtrate was analyzed by Uv-vis spectrophotometer. The concentration obtained is the equilibrium concentration of Cd^2+^ ions in solution (*C*e).

**Figure 3:**
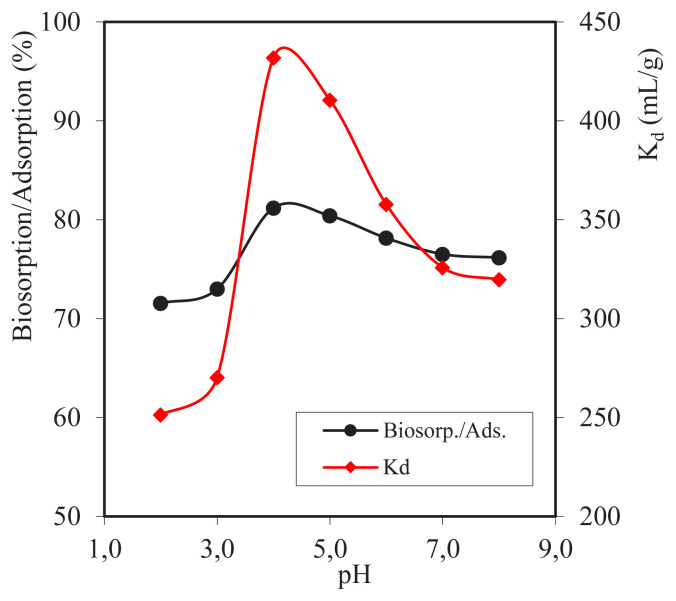
Graph of pH variation on sorption efficiency.

From the calculation results and Figure 3, the optimum pH efficiency obtained is at pH = 4 of 81.20%. At low pH value, the absorption of metal ions is smaller because, at low pH value, the surface of the adsorbent is surrounded by H^+^ ions, so the surface of the adsorbent is positively charged. This can lead to the repulsion of the adsorbent with metal ions. At pH = 5, 6, 7, and 8, there was a slight decrease, i.e., the results obtained were 80,41%, 78,15%, 76,51% and 76,17%; this could be due to the fact that at that pH value, the concentration of the active site on the biosorbent with Cd^2+^ ions was in equilibrium, so Cd^2+^ metal ions that were absorbed decreased slightly [14].

Figure 3 shows an increase in *K*_d_ in the range of pH = 2.0–4.0 and the largest *K*_d_ at pH = 4.0 is 431.94 mL/g; it has a slight decrease at pH = 5.0, 6.0, 7.0, and 8.0 with the *K*_d_ values of 410.43, 357.72, 325.83, and 319.68 mL/g. This happens because of low pH; the concentration of H^+^ ions is higher, so there is a competition with Cd^2+^ metal ions to interact with the active site of functional groups on the adsorbent. While, at pH = 5.0, the number of H^+^ ions began to decrease, the active site on the composite biosorbent absorbed more Cd^2+^ metal ions.

### 3.2 Effect of variation of concentration of Cd^2+^ ion solution

**Figure 4:**
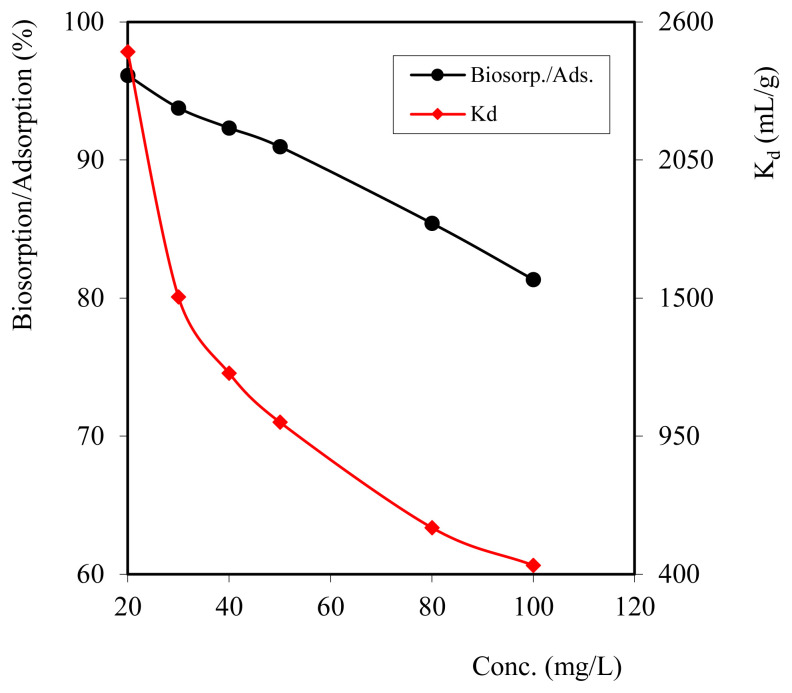
Graph of Cd^2+^ concentration variations on sorption efficiency

This variation of Cd^2+^ metal ion solution is very important in order to know how much concentration of Cd^2+^ metal ion is adsorbed using composite biosorbent. Concentration variations were carried out by varying the Cd^2+^ metal ion solution, namely 20, 30, 40, 50, 80, and 100 mg/L while keeping all the other parameters constant. The results of the effect of concentration variations can be seen in Figure 4. The results of the sorption of Cd^2+^ ions with various concentrations showed that the sorption efficiency decreased with increasing concentration in the solution, but there was a slight decrease at a concentration of 100 mg/L because the biosorbent was completely filled with Cd^2+^ ions, so an equilibrium was achieved between the surface area of composite biosorbent and the number of adsorbed Cd^2+^ metal ions. At sorption equilibrium conditions, the surface of biosorbent has been filled with adsorbate, so that it is no longer able to absorb Cd^2+^ metal ions.

In Figure 4, it can be seen that the *K*_d_ decreases with increasing concentration in the solution, and the higher the concentration of the solution, the higher the sorption capacity. This capacity increase occurs because the number of ions bound to the adsorbent is greater.

### 3.3 Effect of contact time variations

Contact time is the time required for the composite biosorbent to absorb metal ions. Time is also one of the composite biosorbent factors because it takes time to reach the metal equilibrium state by the biosorbent. Variations in contact time in this study were 15, 30, 60, 120, 180, 240, 300, and 360 min with the optimum pH value, and the concentration of the Cd^2+^ metal ion solution was 100 mg/L.

**Figure 5:**
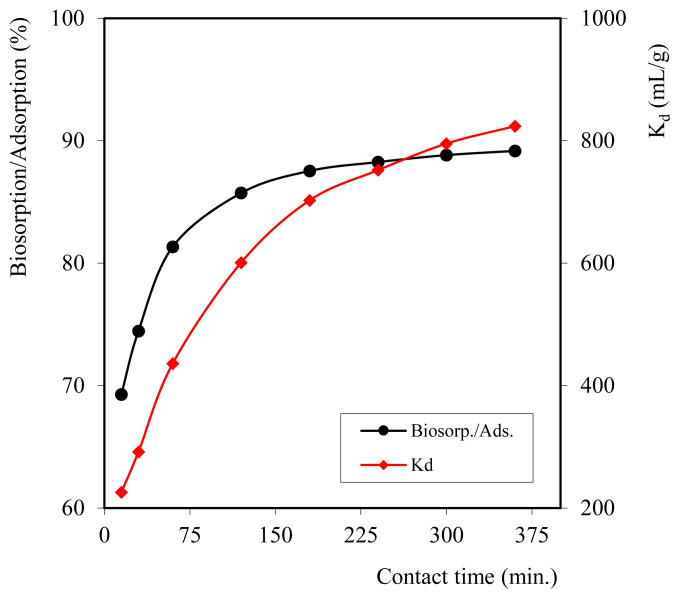
Graph of time variation on sorption efficiency.

Figure 5 shows that the contact time of Cd^2+^ metal ions with composite biosorbent affects the absorption of Cd^2+^ metal ions. The longer the contact time, the greater the sorption of Cd^2+^ metal ions by composite biosorbent. At contact times of 15, 30, 60, 120, and 180 min, the absorption of Cd^2+^ metal ions’ efficiency rates were 69.28%, 74.45%, 81.34%, and 85.73%, respectively. The increase in efficiency continued to occur at contact times of 240, 300, and 360 min. This indicates that as the contact time lasts longer, the sorption will continue to increase. However, the increase in efficiency at contact times of 240, 300, and 360 min is relatively small; it is possible that the amount of adsorbed substance will reach the equilibrium limit.

The results of variations in contact time efficiency and *K*_d_ values were similar, i.e., the distribution coefficient increased over time. The contact time was taken as 60 min to examine the biosorbent amount effect.

### 3.4 Amount effect biosorbent

**Figure 6:**
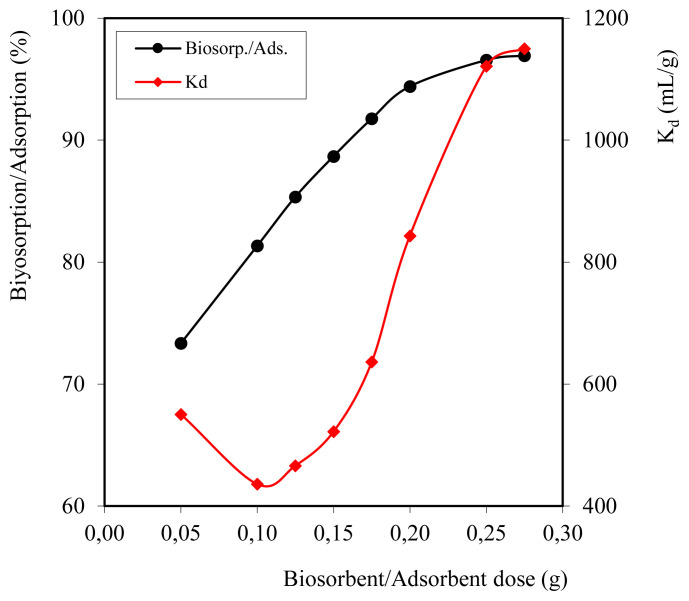
Graph of biosorbent dose variation on sorption efficiency.

Different amounts of the biosorbent (0.05, 0.1, 0.125, 0.150, 0.175, 0.20, and 0.25 g) were used to find the optimum dose required to adsorb maximum adsorbate ions. The results of the effect of adsorbent dosage on the sorption of Cd^2+^ ions are given in Figure 6. It was observed that the sorption process increased with the increase of the amount of biosorbent and became uniform after reaching the optimum dose, i.e, 0.20 g. It was noted that at 0.25 g composed adsorbent amount, 96.93% of Cd^2+^ ions are removed. The *K*_d_ and *Q*_e_ values were calculated as 1149.85 mL/g and 3877.4 mg/g, respectively.

### 3.5. Adsorption isotherms

Adsorption isotherm is a method used to describe a state of equilibrium between the concentration of solutes adsorbed on the solid surface and the amount of absorption at a constant temperature. Adsorption equilibrium can occur when the concentration of metal ions absorbed is equal to the concentration of metal ions that come out. The interaction between the adsorbent and the adsorbate can be described by several types of adsorption isotherms including Freundlich adsorption isotherms, Langmuir adsorption isotherms, and Dubinin and Raidushkevich (D-R) isotherms. These three adsorption isotherms are the types of isotherms commonly used for adsorption in the solid-liquid phase. If the data obtained are a Freundlich adsorption isotherm, then the adsorbent used has a heterogeneous surface and is multilayer in nature [15]. Meanwhile, if the data obtained are a Langmuir adsorption isotherm, then the adsorbent used has a homogeneous surface and is a monolayer [16].

The adsorption model that is often used to determine the adsorption equilibrium is the Langmuir and Freundlich isothermal [17]. Eq. (4) below is the Langmuir isothermal equation.


(4) $$q_{e}=\frac{q_{max}.bC_{e}}{1+bC_{e}}$$

where *q*_max_ is the maximum adsorption capacity associated with the total monolayer adsorption coverage (mg adsorbate/g adsorbate), and *b* is the Langmuir constant associated with the adsorption energy (*L* adsorbate/mg adsorbate).

Eq. (4) can be linearized [18] as follows:


(5) $$\frac{1}{q_{e}}=\frac{1+bC_{e}}{q_{max}.bC_{e}}$$

(6) $$\frac{C_{e}}{q_{e}}=\frac{1}{q_{max}.b}+\frac{1}{q_{max}}C_{e}$$

(7) Y=a+bX

Freundlich developed the adsorption isotherm by assuming that the surface of the adsorbent is heterogeneous, and this model is suitable for use in aqueous and mixed solutions. The Freundlich equation used [19]:


(8) $$q_{e}=k_{f}C_{e}^{1/n}$$

where *k_f_* is the Freundlich constant associated with the adsorption energy (L biosorbent/mg adsorbate), and *n* is the empirical constant. The linearization of Eq. (8) is:


(9) $$log\;q_{e}=log\;k_{f}+\frac{1}{n}logC_{e}$$

*k_f_* is important in determining the adsorption capacity, which arises from the influence of most of the properties of the adsorbent.

The adsorption capacity of the adsorbent varies according to the pH value and temperature of the solution, as well as the pore, particle size, specific surface area, cation exchange capacity, and functional group surfaces of the adsorbent material.

As seen in Eq. (9), the Freundlich constant *k*_f_ and *n* values can be calculated from the intercept and slope, respectively, in the log *q*_e_ versus log *C*_e_ graph. The value of *k*_f_ depends on the units *q*_e_ and *C*_e_. If 1/n is 1.0 then *k*_f_ is equal to *q*_e_ when *C*_e_ is equal to 1.0. In this case, if 1/*n* is equal to one, the adsorption energy is identical for all sites [20,21]. Stronger adsorbent-adsorbate interactions usually occur at a larger n value. Freundlich is suitable for isotherm of adsorption if the value of n has a value in the range of 1 to 10 [22].

D-R adsorption isotherms are generally used to explain sorption isotherms that occur in micropores [23].

This isotherm, which is used to predict the pore structure of the adsorbent and the properties of adsorption, is used to calculate the inhomogeneous surface or fixed adsorption potential [24].

In addition, the free energy of adsorption can be determined by this model. The linear equation of the D-R adsorption isotherm model is given below [25]:


(10) $$q_{e}=Q_{m}\;\text{exp }(K{ɛ}^{2})$$

where *Q*_m_, *K*, and ɛ are the adsorption capacity of the adsorbent per unit mass, the affinity coefficient and the constants associated with the adsorption energy, and the temperature-related Polanyi adsorption potential, respectively.

The D-R equation can also be expressed with a linear equation [26]:


(11) $$lnq_{e}=lnQ_{m}-K{ɛ}^{2}$$

When ɛ^2^ values versus ln*q*_e_ values in Eq. (11) are plotted, *K* constant and adsorption capacity can be determined from the slope and intersection point, respectively. The constant *K* is defined as 1/ *βE* in relation to the affinity coefficient (*β*).

The average free energy value *E*(kJ/mol) per adsorbate molecule used to determine the adsorption properties can be calculated by the equation given below [27].


(12) $$E=\frac{1}{\sqrt{2K}}$$

The *E* value is commonly used to make an estimation of the nature of physical or chemical adsorption processes [28].

In addition, the average free energy can be calculated by the following equation:


(13) $$ɛ=RTln\;\left(1+\frac{1}{C_{e}}\right)$$

The D-R isotherm can be used not only for one adsorbate but also for a two-adsorbate system [29]. The assumption of this model is that adsorption has a multilayer character, involves van der Waals forces, and is applied for physical adsorption [30,31]. The D-R isotherm is only suitable for a medium range of adsorbate concentrations.

**Figure 7:**
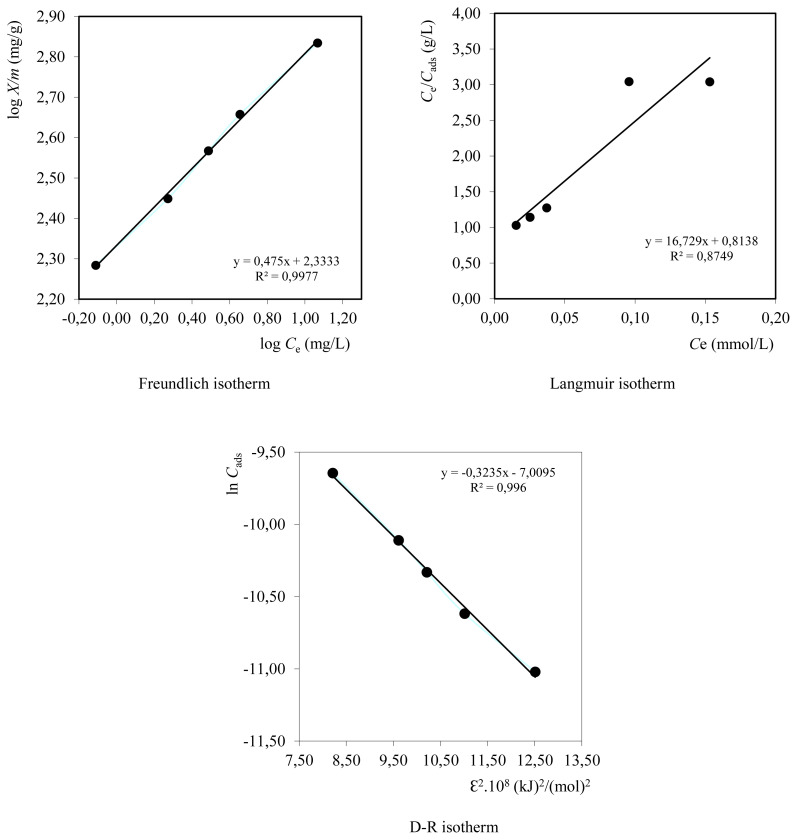
Freundlich, Langmuir, and D-R isotherms graphs.

The determination of the Cd^2+^ metal ion adsorption isotherm model is shown in Figure 7. The Freundlich isotherm curve is the relationship curve between log *C*e and log *X/m*, where log *C*e is the log of concentration after equilibrium, and log *X/m* is the log of adsorbate mass absorbed per *g* biosorbent.

From the three adsorption isotherm curves, the Cd^2+^ metal ion adsorption model can be determined by composite biosorbent by comparing the linear regression coefficient (*R*^2^) of the adsorption isotherm curve, namely 0.9977 for the Freundlich adsorption isotherm, 0.8749 for the Langmuir adsorption isotherm, and 0.9960 for the D-R adsorption isotherm. The correlation coefficient (*R*^2^) of the Freundlich and D-R isothermal is greater than that of the Langmuir isothermal, which are 0.9977 and 0.9960. This shows that there is a good correlation between the experimental data and the mathematical model equation for the Freundlich and D-R isothermal. The metal ion adsorption mechanism of Cd^2+^ by composite biosorbent follows the Freundlich and D-R adsorption isotherm model. The data obtained from the three isotherms are given in Table 2.

As seen in Table 2, the values of Freundlich constant *K*_f_ and *n* coefficients were found as 215.3 (mg/g) and 2.04, respectively. These values indicate the sorption density and sorption capacity, respectively. The Freundlich sorption isotherm cannot predict the presence of sites on the surface that are able to prevent sorption when equilibrium is reached, and there are only a few active sites that are able to adsorb dissolved molecules. The mechanism that occurs in the Freundlich sorption isotherm is physisorption.

The DR isotherm is used to estimate the mean free energy of the adsorption event (*E*). If the *E* value is between 1.0 and 16.0 kJ/mol, adsorption is physical, and if the value is more than 16.0 kJ/mol, it is chemisorption [25,32]. As shown in Table 2, the *E* values are 12.432 kJ/mol for Cd^2+^ on the composite biosorbent. The value of *E* is expected for chemical sorption. It is assumed that the structure of the composite biosorbent containing Na-alginate is heterogeneous.

### 3.6. Thermodynamic parameters

Thermodynamic parameters are important aspects in sorption studies; these parameters provide a better explanation of the effect of temperature on the sorption process. Thermodynamics for Cd^2+^ ion transfer with composite biosorbent were investigated in a certain temperature range. In the current study, the temperature range was between 283.15 K and 308.15 K. Thermodynamic parameters: enthalpy change (Δ*H*^o^), and entropy change (Δ*S*^o^) can be determined by the following Eq (14):

Thermodynamics for the sorption of Cd^2+^ ions with composite adsorbent, with the following Eq. (14):


(14) $$Ln\;K_{d}=\left(\frac{\Delta S}{T}\right)-\left(\frac{\Delta H}{RT}\right)$$

Gibbs free energy (ΔG) was calculated by using the following well-known Eq (15).


(15) ΔG=ΔH-TΔS

Thermodynamic analysis was carried out to determine the enthalpy (ΔH), entropy (ΔS), and Gibbs free energy (Δ*G*) obtained by plotting ln *K*_d_ vs. 1/*T* at each temperature. The thermodynamic parameters on the sorption of Cd^2+^ metal ions with composite biosorbent are shown in Table 3. The values of Δ*S^o^* (kJ/mol K) and ΔH^o^ (kJ/mol) were calculated from the slope and intercept of the graph (Table 3).

Table 3 shows that Δ*G*° sorption of Cd^2+^ ions with composite biosorbent have a negative value at each sorption temperature. This phenomenon indicates that the adsorption process runs spontaneously [33].

The negative value of Δ*G*°, which is quite large, explains that energetically, the sorption process is preferable to other sorption mechanisms. The value of Δ*G*° in this study decreased with increasing temperature, it showed that, at higher temperatures, there was a decrease in the driving force, which resulted in a decrease in the sorption process of adsorbate molecules on the biosorbent. This is in accordance with the results obtained where the sorption capacity of Cd^2+^ ions using composite biosorbent decreases with increasing sorption temperature.

The positive value for Δ*S*° indicates an increase in entropy that occurs at the solid-solution interface during the sorption process. This positive value of ΔS° indicates that the sorption process also tends to occur spontaneously as indicated by the Gibbs free energy value. The positive Δ*S* value suggested an increase in molecular disorder at higher temperatures [34,35]. The results of this study are in accordance with the research results of Mahmood et al. [36] who also found that the adsorption of cadmium using alginate–calcium carbonate composite beads also had a value of Δ*S*°>0.

The value of Δ*H°* obtained from this research is −44.91 kJ/mol. A negative value of Δ*H°* was obtained on the sorption of Cd^2+^ ions with composite biosorbent, which indicated that the adsorption process was exothermic [37]. The amount of sorption energy can determine whether the sorption process follows the chemisorption or physisorption process. The chemisorption process involves a direct bond between the adsorbate and the adsorbent surface. Physisorption does not involve direct bonding. This value indicates that the process that occurs in the sorption of Cd^2+^ for composite biosorbent is a physisorption process [38].

## 4. Conclusion

The sorption test for synthetic composite biosorbent showed that the optimum contact time obtained was 60 min, the concentration was 8.0913 mg/L, the temperature was 25^o^C, and the optimum pH value for sorption was 4.0. The sorption equilibrium model can be represented by the Freundlich equilibrium model with an *R*^2^ value, which is 0.9977. The type of sorption used in this study is physical sorption (physisorption), which is characterized by a low and negative ΔH° value, namely −44.91 kJ/mol and Δ*S*° = 0.203 kJ/mol K. The negative Δ*H°* value indicates that the reaction is exothermic. The value of Gibbs free energy (ΔG°) is negative, −105.43 kJ/mol, so that it can be concluded that the reaction is spontaneous.

For further research, the sorption kinetics of the composite biosorbent to Cd^2+^ metal can be determined, and it can be applied to deal with pollution problems in various industries.

## Figures and Tables

**Table 1:** Percent mixing ratios of the composite biosorbent composition.

Composite biosorbent composition	%
Diatomite	59.41
Jibs	17.29
Na-alginate	10.41
Tetra sodium pyrophosphate	7.61
Magnesium oxide	4.94
Potassium fluoro titanate	0.334
Total	100

**Table 2:** Parameters of Freundlich, Langmuir and D-R isotherms for sorption of Cd^2+^ metal ions on composite biosorbent.

Langmuir parameters		Freundlich parameter		D-R parameters		
*Q*(mmol/g)	b(L/g)	*K*_f_ (mg/g)	*n*	*X*_m_ (mmol/g)	*β* (mol^2^/J^2^)	E (kJ/mol)
0.378	8.973	215.3	2.10	0.90	0.32×10^−8^	12.432

**Table 3:** Thermodynamic parameters of Cd^2+^ ions sorption using composite biosorbent.

Equality	ΔH° (kJ/mol)	ΔS° (kJ/mol)	*R* ^2^	ΔG° (kJ/mol)				
				288.15	293.15	298.15	303.15	308.15
y = −5401.6× + 24.412	−44.91	0.203	0.9953	−103.40	−104.42	−105.43	−106.45	−107.46
